# Molecular pathways in the development of HPV-induced cervical cancer

**DOI:** 10.17179/excli2021-3365

**Published:** 2021-02-12

**Authors:** Farnaz Rasi Bonab, Amir Baghbanzadeh, Moslem Ghaseminia, Nadia Bolandi, Ahad Mokhtarzadeh, Mohammad Amini, Kianoosh Dadashzadeh, Khalil Hajiasgharzadeh, Behzad Baradaran, Hossein Bannazadeh Baghi

**Affiliations:** 1 Immunology Research Center, Tabriz University of Medical Sciences, Tabriz, Iran; 2 Department of Laboratory Sciences, Marand Branch, Islamic Azad University, Marand, Iran; 3 Department of Virology, Faculty of Medicine, Tabriz University of Medical Sciences, Tabriz, Iran; 4 Department of Immunology, Faculty of Medicine, Tabriz University of Medical Sciences, Tabriz, Iran

**Keywords:** cervical cancer, HPV, oncoprotein, E6 and E7, signaling pathway

## Abstract

Recently, human papillomavirus (HPV) has gained considerable attention in cervical cancer research studies. It is one of the most important sexually transmitted diseases that can affect 160 to 289 out of 10000 persons every year. Due to the infectious nature of this virus, HPV can be considered a serious threat. The knowledge of viral structure, especially for viral oncoproteins like E6, E7, and their role in causing cancer is very important. This virus has different paths (PI3K/Akt, Wnt/β-catenin, ERK/MAPK, and JAK/STAT) that are involved in the transmission of signaling paths through active molecules like MEK (pMEK), ERK (pERK), and Akt (pAkt). It's eventually through these paths that cancer is developed. Precise knowledge of these paths and their signals give us the prognosis to adopt appropriate goals for prevention and control of these series of cancer.

## Introduction

**Figure 1:**
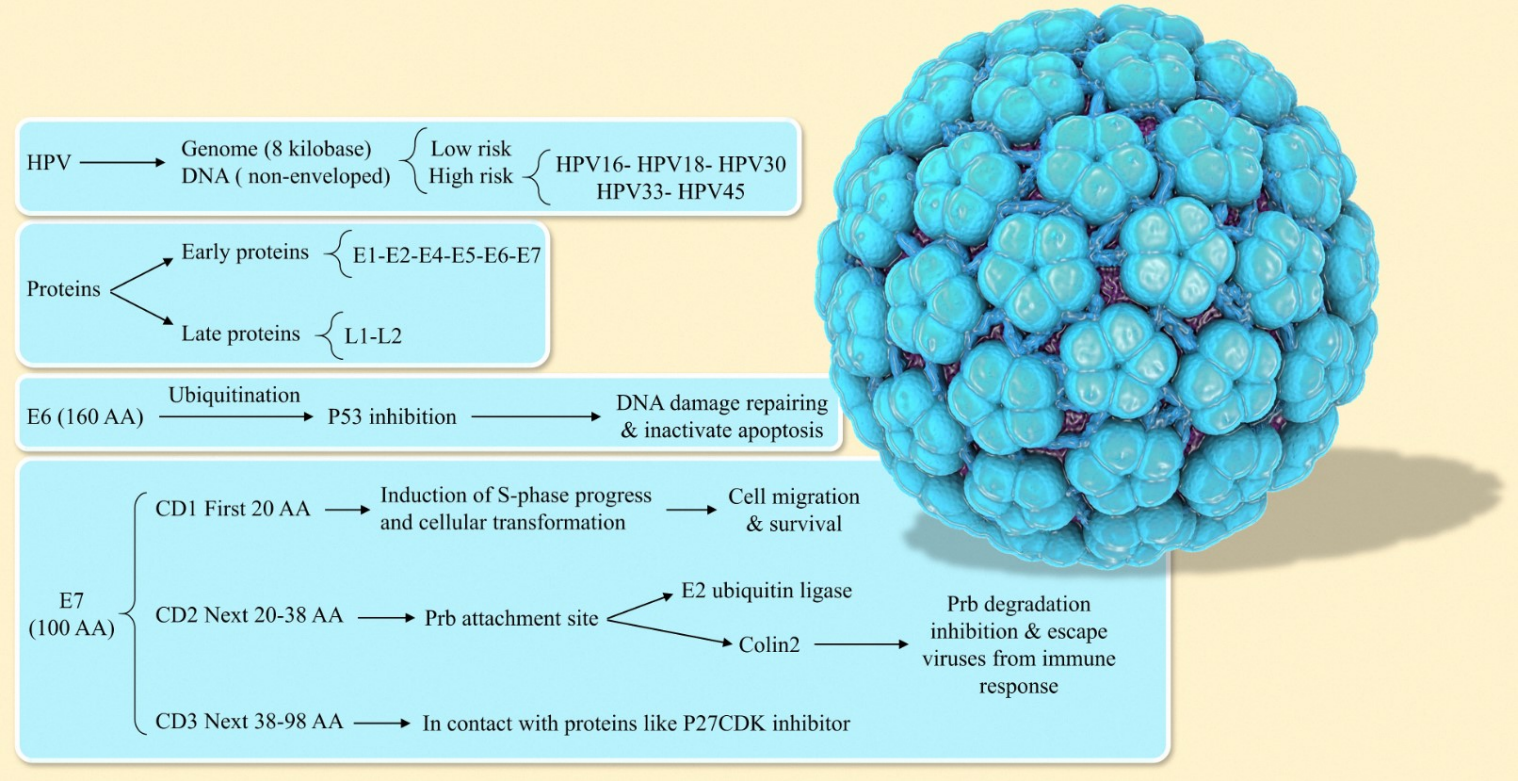
There is a strong association between human papilloma virus (HPV) infection and the development of human cervical cancer. E6 and E7 proteins of HPV have a pivotal role in the initiation and progression of HPV-associated cervical cancer.

Cervical cancer is the commonest occurring cancer in women. In underdeveloped countries, the incidence of cervical cancer is high (about 85 percent) (Manikandan et al., 2019[100]). Cervical cancer ranks second as a cause of cancer-specific mortality in women (Aaronson, 2002[1]; Jemal et al., 2011[80]; Li et al., 2015[96]). The most prominent types of cancer among women are breast, colorectal, gastric, oral, lung, and cervix (Hoque and Hoque, 2009[72]). Human papillomavirus (HPV) is the most well-known risk factor for the development of cervical cancer. Several types of viruses can cause cervix infection. Studies show that 97.7 % of cervix cancer patients are HPV positive. At the present time, HPV of types 16, 18, and 45 have been identified as the most common causes of cancers of the cervix (Blodt et al., 2012[12]; Pappa et al., 2017[117]). This virus can cause cancer not only in the cervix, anus, penis, vulva, and vagina but also in some other types of head and neck cancers (Nour, 2009[108]). In the first stages of cancer, the basal layer cells get infected, and if prolonged, the HPV genome survives as a nuclear extrachromosomal episome by suppressing the viral oncoproteins E6 and E7, and following gradual basal cellular differentiation. Besides, basal cellular migrates toward the higher epithelium layers. As a result, there is an increase in the expression of viral oncoproteins E6 and E7 together, which leads to inhibition of apoptosis and proliferation of the virus-derived genome (Kontostathi et al., 2016[88]). The majority of HPV infections could display no signs of disease in patients for the first few years and might disappear with no treatment (Manikandan et al., 2019[100]). In this review, we will focus on the effect of HPV and the types of proteins that facilitate the evolution of HPV infection into cancer HPV structure. Our goal in this paper is not to give a full description of the HPV structure that has been covered in all scientific sources. We are only discussing some general facts and then move to the details. Regarding structure related to HPV, it has been demonstrated that the HPV as a deoxyribonucleic acid (DNA) virus has a non-enveloped structure which causes papillomatoses in a variety of vertebrates. Its genome has a circular shape of size of approximately 8 kb. This virus, depending on its potential for causing cancer, is called low-risk or high-risk HPV. Among these viruses, types 16, 18, 30, 33, and 45 are considered the most dangerous. Each virus has 8 principal proteins. In the early region of the HPV genome, there are 6 proteins while the late region encodes 2 proteins (Doorbar et al., 1997[37]; de Sanjose et al., 2010[29]; Graham, 2010[55]). The proteins in E3, E8 have also been identified, that are involved in protein-coding of HPV-31 and BPV-1 (Zheng and Baker, 2006[170]) (Figure 1(Fig. 1)).

## Early Proteins

These proteins are among functional proteins that are involved not only in the control of infected cells via apoptosis but also in the increase of virus genome replication; transcription, cell signaling, cell cycle, immune modulation, and structural modification (Doorbar et al., 1997[37]).

### E1

E1 is a viral DNA helicase (Bergvall et al., 2013[10]). This protein, in tandem with E2, can regulate the immediate-early transcription and also contributes to viral DNA (Zheng and Baker, 2006[170]). E1 is not only an enzyme but also the most protected protein that is coded by Papillomavirus (PVs). It appears that E1 is needed in the proliferation cycle of viral because of its significant role revealed in the viral cycle. Initially, keratinocyte infection causes an increase in the number of viral episomes copies and then the distinction begins with an increase in the constant level of the episodes. Finally, in the upper layers of the epithelial, strengthening the viral genome occurs during stages of life cycle production related to the virus (Kim and Lambert, 2002[85]; Egawa et al., 2012[42]; Bergvall et al., 2013[10]).

### E2

E2 has a second -N terminal that acts as a conduit for inter-protein reactions, a flexible hinge, as well as a second connection to C-terminal DNA (Stubenrauch et al., 2000[145]; Ammermann et al., 2008[3]). It seems that the expression of E1 and E2 proteins occur in small quantities in the prior stages of basal layer cells of infection (Ozbun, 2002[115]). Studies have shown, that these proteins act as repressors for P97 transcription after TATA-binding protein (TBP), by connecting TFIID (Hou et al., 2000[73]). Also, transcriptional repression only occurs in the cells that have uniform DNA (Stanley, 2006[144]). In high-risk HPV and mainly in HPV16 and HPV17, the original transcribed copy starts from a promoter named P97 (Bechtold et al., 2003[8]; Stanley, 2006[144]). This protein has an important role in controlling the transcription of oncogenes such as E6/E7, and viral DNA during the viral life cycle (Bergvall et al., 2013[10]). 

### E4

E4 coding sequences are included in E2 (ORF). Although, E4 is in the early region its expression is realized in the late region. Furthermore, the properties of E4 are not completely identified, but some researchers conclude that E4 is involved in viral spread through keratin filaments. This protein is differentiated in keratinocytes and occurs in the collapse of cytokeratin filament in conjunction with generative infection (Smith et al., 2007[140]).

### E5

E5 Expression causes the process of cell proliferation to be enhanced. Besides, expression of E5 induces reinforcement of genome in the HPV-16 and HPV-31 life cycle production stage (Wechsler et al., 2018[162]). Previous studies have shown that the E5 Expression could disappear following the merger of the HPV genome in the host chromosome, but many studies show that E5 transcripts can be positive in ovaries cancer biopsies (DiMaio and Mattoon, 2001[35]). This oncoprotein in HPV-16 can directly connect to the vacuolar ATPase (V-ATPase) 16-kD subunit, and control the activity of V-ATPase and eventually change the EGF receptor (Schwarz et al., 1985[135]; Um et al., 2014[154]), whose overproduction can cause tumor formation (Kim et al., 2010[86]).

### E6

E6 is one of the most important HPV oncoproteins that can cause transformations in the host cells. This oncogene protein targets the p53 protein, and by suppressing it causes the development of cancer tumors and the inhibition of apoptosis (Ruttkay-Nedecky et al., 2013[131]; Mesri et al., 2014[103]). E6 has 160 amino acids, contributes to P53 inhibition through ubiquitination (Kruiswijk et al., 2015[89]; Paek et al., 2016[116]). P53 is the simulation agent of genes that not only repairs the DNA that has been damaged in the life cycle of a cell but also activates the path of apoptosis (Paek et al., 2016[116]). The mdm2 pathway is entirely inactivated in cancerous cells (Hengstermann et al., 2001[64]). Furthermore, the regulation of p53 function is accomplished by E6. It also has a role in the transfer of the p53 gene (Pim et al., 1994[123]). E6 can control the dependent gene of p53 by the reaction to p300/CBP (Patel, 1999[119]; Zimmermann et al., 1999[173]). The acetylation inhibition of core histones in nucleosome and p53 correlated with the suppression of the activity related to p53 which is mediated by E6. Accordingly, the p300 and p53 have been altered to chromatin (Thomas and Chiang, 2005[149]; Tomaić, 2016[152]). Moreover, this oncogene protein has the ability to target the pro-apoptotic proteins like Bak. Consequently, the apoptosis process is inhibited (Thomas and Banks, 1998[148], 1999[147]; Jackson and Storey 2000[78]). Also, survivin has been identified as the indirect target of E6 for inhibiting apoptosis (Borbély et al., 2006[13]). This oncoprotein interacts directly with other components of apoptosis including TNF R1 (Necrosis factor receptor 1 tumor), Fas-associated protein with death domain, and procaspase-8 (Filippova et al., 2002[46], 2004[45], 2007[44]).

### E7 

E7 is another important oncoprotein that has a complementary function to Retinoblastoma protein (PRb). Expression of E7 resulted in E2F is released and stimulation of DNA synthesis (Yim and Park, 2005[167]; Ganguly and Parihar, 2009[51]). Its length is about 100 amino acids (Tommasino, 2014[153]). CD1, CD2, and CD3 are 3 domains related to E7 which are conserved with the most important functions in CD2 and CD3 (Patrick et al., 1994[120]). Transformation of cellular and induction of S-phase progression is the result of E7 function and the presence of the first twenty amino acids sequence in the CD1 domain (Banks et al., 1990[6]; Demers et al., 1996[32]). The two binding partners, UBR4/ p600, and p300/CBP-associated factor (P/CAF) interact with CD1. They are crucial for membrane morphogenesis and cellular survival (Huang and McCance, 2002[74]; DeMasi et al., 2005[31]; Huh et al., 2005[76]; Nakatani et al., 200[107]5; White et al., 2012[163]). Members of the NF-kappaB (NF-κB) family are inactivated by E7 and P/CAF that happens during viral infection and causes the immune system to no response to the virus (Huang and McCance, 2002[74]). The CD2 region of the E7 has been constituted of the amino acid residue of 20-38. This includes the LXCXE motif and site of CKII phosphorylation, which are incorporated in binding to proteins like the retinoblastoma tumor suppressor protein (pRb). Thus, the site of the CKII phosphorylation acceptor is significant for the transforming-capacity of E7 (Barbosa et al., 1990[7]; Firzlaff et al., 1991[47]). E7 is interesting for its contribution to the adjustment of DNA methylation for controlling cell proliferation routes and can cause epigenetic modifications using Rb as a tumor suppressor protein (Dueñas-González et al., 2005[38]). Cullin2 ubiquitin ligase in conjunction with E7 oncoprotein can degrade tumor suppressor pRb. Hence, the progression of the cell cycle is promoted (Huh et al., 2007[75]). The transition of the phase from G1 to S as well as the cell cycle is regulated by pRb. In a normal situation, phosphorylation of pRb gradually takes place during S-phase, but not in the early G1-phase. Besides, the interaction of unphosphorylated form of the pRb with the factors involved in transcription such as E2F resulted in suppression of transcription of E2F sites in promoters (Dyson et al., 1989[41]). The C-terminus, which refers to the region of CD3, consists of amino acid residues from 38 to 98 and 4 cysteine residues that have high conservation of cysteine and can interact with inhibitors of p27 CDK and p21. Therefore, the abrogation of E7 activity is an important step in two phenomena of (I) overcoming the cell cycle arrest that includes the damage of DNA and (II) inducing the progress of the cell cycle (Jones et al., 1997[81]; Helt and Galloway, 2001[63]).

## Late Proteins

This region with 850 bp size covers about 40 % of the virus genome and contains 10 percent of the HPV genome. The late region, with several binding sites of the transcription factor, can regulate RNA polymerase II at the beginning of the transcription also; replication of the HPV genome is controlled by this region. Furthermore, it is noteworthy that the late region can't encode proteins (Zheng and Baker, 2006[170]).

### L1

L1 is the most fundamental capsid protein that is produced during the cellular life cycle in the cytoplasm and then moves to the core. In the top layer of epithelial, 360 L1 as a capsid protein can encapsulate the viral DNA to make novel particles of infective viral. Besides, the completion of the HPV life cycle is determined by the attendance of L1 capsid protein in the dysplastic cells (Griesser et al., 2009[56]). It seems that the high-grade squamous intraepithelial lesions (HSIL) are not capable of producing L1 protein, and this can be used as a prognosis for detecting CIN damages (Stanley, 2006[144]).

### L2

L2 appears as a small part of the capsid and plays a role in viral DNA classification and montage (Buck et al., 2008[17]).

## HPV Oncoproteins and Signaling Pathways

**Figure 2:**
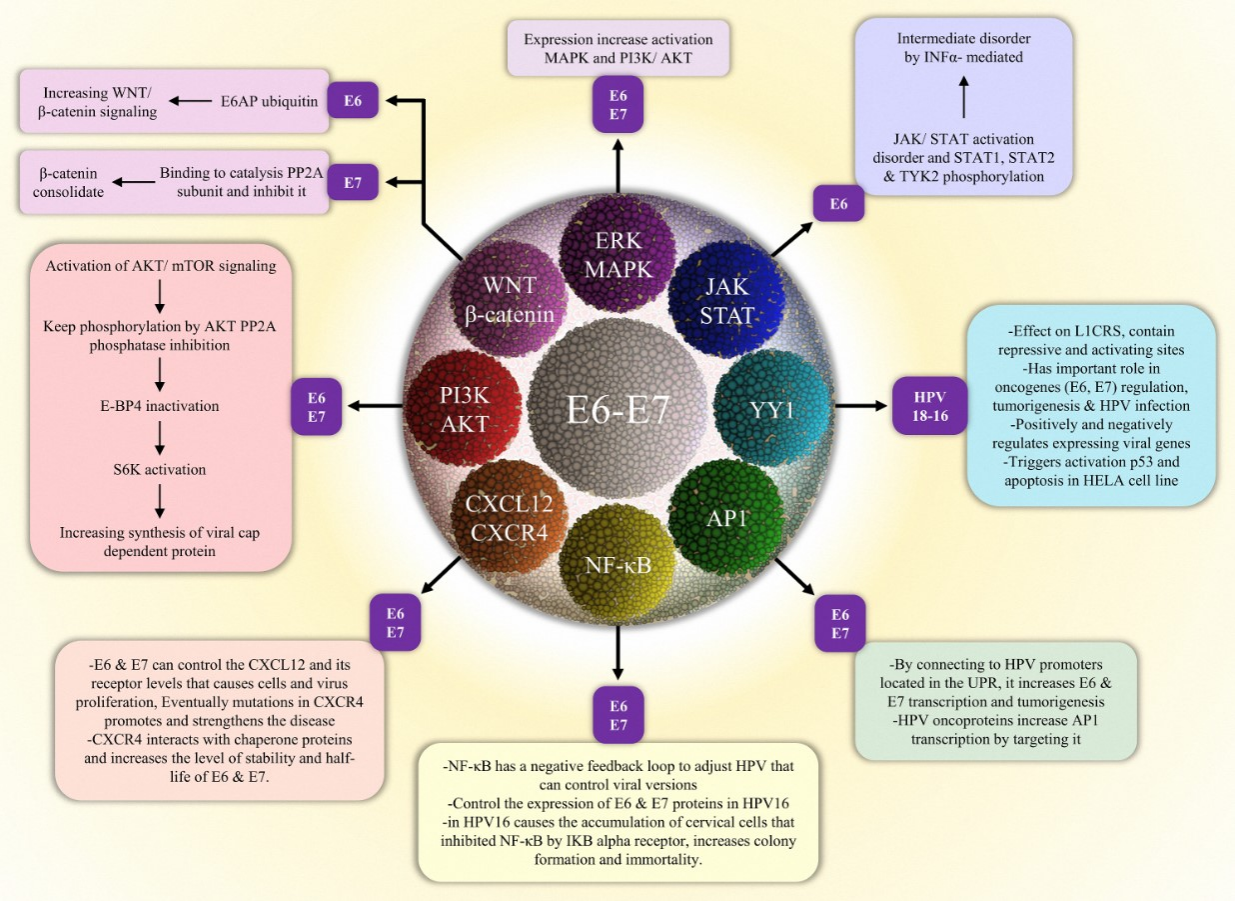
The role of E6/E7 oncoproteins in HPV-associated cervical cancer development. E6 and E7 react with numerous intracellular signaling pathways, resulting in induced carcinogenesis.

Extracellular stimuli are converted into cellular response through signal pathways, starting from a signal to a receptor, and changing the cellular function. Occasionally there is a cascade of amplifying signals, resulting in a large response (Campbell et al., 2000[21]). Such a signal produces changes in the cell function and results in the changing expression of the genes in the nucleus or in the activity of the enzymes. Most of these signals create bonding between extracellular molecules to cell surface receptors (Campbell et al., 2000[21]). Defects in signaling pathways are the leading cause of different diseases like diabetes, autoimmune diseases, heart disease, and cancer (Costa et al., 2010[28]). HPV can use different mechanisms to evade the proper autoimmune response of the host, through its oncoproteins (Tindle, 2002[151]). During infection two proteins (E6 and E7) are involved in the process of pathogenicity (Androphy et al., 1987[5]; Mesri et al., 2014[103]; Zhang et al., 2019[169]) (Figure 2(Fig. 2)). 

Apoptosis is a genetically controlled cell self-destruction process whose function is essential for cellular growth, cellular homeostasis, and the pathophysiology of various diseases (Rudin and Thompson, 1997[130]; Shirjang et al., 2019[138]). Apoptosis is controlled by two pathways (Lopez and Tait, 2015[98]). Abnormal proliferation, as well as DNA damage, are some of the factors that cause activation of the apoptosis signaling pathway (Ruttkay-Nedecky et al., 2013[131]). In the next subsections, we discuss in more detail the role of HPV oncoproteins and their signaling pathways in the development of HPV-associated cervical cancer (Table 1(Tab. 1)).

### HPV and PI3K/Akt pathway

PI3K activity is physically and functionally associated with the activity of cellular processes such as protein synthesis and metabolism of glucose (Whitman et al., 1985[164]). PI3K is divided into 3 separate classes based on their structure, configuration, and performance (Vanhaesebroeck et al., 2012[158]). The IA class of heterodimer consists of 3 monitoring subunits, including p85a, p85b, or p55c, which is essentially confined to a p110a, p110b, or p110d subcatalyzed isoform. The conversion of phosphatidylinositol (4,5)-bisphosphate (PIP2) to phosphatidylinositol (3,4,5) triphosphate (PIP3) occurs when the interaction of regulatory subunit with the Receptor tyrosine kinases (RTK) or G-protein-coupled is activated by the catalyst subunit. Ras can also directly activate the catalyst subunit. Phosphatase and tensin homolog (PTEN) can negatively regulate the PIP3 and when activated, domain-containing proteins the Pleckstrin Homology (pH) is attracted to the cell membrane, one of which is a kinase B protein called Akt. AKT is phosphorylated on Ser473 and Thr308 via DNK-PK and PDK1, respectively. Activation of Akt causes phosphorylation of nuclear factor-κB (NF-κB), TSC2 (tuberous sclerosis complex 2), FOXO transcription factors (forkhead-box type O), GSK3b (glycogen synthase kinase-3b), MDM2 (murine double minute 2), and Bcl2 cell agonists. Furthermore, mTOR complex 1 (mTORC1) is activated when tuberous sclerosis complex2 (TSC2) is phosphorylated via AKT (Kang et al., 2008[82]; Burris, 2013[18]). This metabolic pathway controls various cellular processes such as glucose metabolism, proliferation and protein synthesis. Inactivity of this signaling could result in apoptosis. In a viral infection, apoptosis is an effective way to destroy viruses. The virus for its replication needs the activity of the PI3k/Akt pathway to delay apoptosis or prevent apoptosis activation (Galluzzi et al., 2008[50]). In response to this cellular action, this pathway acts as a strategic defense against viral attack (Chang et al., 2006[23]; Kaur et al., 2008[84]; Freudenburg et al., 2010[48]). Both the E6 and E7 maintain the status of the phosphorylation through Akt phosphatase PP2A inhibition and also activate the signaling of Akt/mTOR. By this, not only 4E-BP is inactivated but also S6K is activated which supports the synthesis of viral cap-dependent protein (Spangle and Münger, 2010[143]; Diehl and Schaal, 2013[34]). AKT, as a significant factor in the hypoxia response, is considered not only a mechanistic target of rapamycin (mTOR)/AKT signaling pathway, but also a fundamental member of phosphoinositide 3-kinase (PI3K) (Manning and Toker, 2017[101]). Previous studies demonstrated that when cervical carcinoma cells are cultured under hypoxia, increased activity of PI3K/mTORC2/ AKT signaling pathway can mediate suppression of HPV oncoproteins like E6 and E7 (Hoppe-Seyler et al., 2017[71]; Bossler et al., 2019[15]). E6 and E7 activate these pathways and eventually lead to carcinogenesis (Gupta et al., 2018[58]). Expression of HPV16 oncoprotein E7 affects the activity of Akt and causes an increase in keratinocyte migration associated with P13K/Akt (Charette and McCance, 2007[25]; Anderson et al., 2013[4]).

### HPV and Wnt/β-catenin pathway

The presence of the Wnt family is very important in countless processes, including animal growth and tissue homeostasis in adult organisms. Its proteins are secreted from cells on target cells through a pathway that is very unusual and controlled compared to other signaling pathways. Wnt's defects cause different diseases such as cancer, and degenerative diseases (Willert et al., 2003[165]; Nusse, 2005[110]). The Wnt pathway is a well-known cascade that its function has been specified in the development of primordial germ cells (PGCs); including migration, proliferation, and specification (Kimura et al., 2006[87]; Ohinata et al., 2009[113]; Laird et al., 2011[92]; Chawengsaksophak et al., 2012[26]; Lee et al., 2016[94]). In many developmental processes, the presence of the Wnt/β-catenin signaling cascade is essential. Besides, this signaling pathway is associated with the development of several types of cancer. The signaling starts through the binding of WNT ligands to the complex of the Frizzled/LRP receptor. Wnt secretion is entirely dependent on the acyltransferase Porcupine (Herr and Basler, 2012[65]; Zimmerli et al., 2017[172]). This binding initiates a sequence of effects, which leads to cytoplasmic stabilization and β-Catenin translocation (Nusse and Clevers, 2017[110]; Zimmerli et al., 2018[171]). Significantly, there are 2 pivotal roles of β-Catenin in cells. One of these roles is to act as the key effector in canonical Wnt/β-catenin signaling which is considered the main reason for transducing signals to the nucleus. Consequently, target genes are expressed (Sato et al., 2004[133]; Fu et al., 2011[49]; Polakis, 2012[124]; Valenta et al., 2012[155]). Compared with E6, the role of the E7 oncoprotein in regulating Wnt signaling has not been investigated. Despite that, lately, E7 oncoprotein involvement in this pathway was suggested (Bello et al., 2015[9]). Previous studies show the ability of HPV 16 E6 to cooperate with E6AP in Wnt/β-catenin signaling enhancement or stimulation. Also, transcription of Wnt/β-catenin is improved via the cooperation of E6 with the E6AP in the cells which are activated by Wnt. E6AP, on its own, can not only stabilize β-catenin but also stimulate signaling of Wnt relying on the activity of E3 ligase (Bzhalava et al., 2013[20]; Sominsky et al., 2014[141], 2017[142]; Kuslansky et al., 2016[91]). Studies have shown similar functions of E7 and somatic mutation theory (smt); they both connect to the catalytic subunit of PP2A to impede its activity. This function of E7 might contribute to cytoplasm stabilization (Pim et al., 2005[122]).

### HPV and ERK/MAPK pathway

The MAPK/ERK pathway contains different groups of kinase proteins that transfer extracellular signals to the nucleus. When a tyrosine kinase receptor becomes activated, it stimulates the activity of a MAPKKK (Raf) through the G protein Ras. In turn, it activates the MAPKK (MEK) and eventually, MAPK (ERK) (Yousefi et al., 2012[168]). Some proteins, such as chromatin remodeling, numerous transcription factors that regulate cytoskeletal proteins are phosphorylated by ERK 1/2 (Hilger et al., 2002[66]; Roy, 2002[129]). Cellular machinery and extracellular signal communications are confirmed by pathways mediated by MAPK. In this regard, cellular machinery controls apoptosis, migration, differentiation, proliferation, and growth (Mansoori et al., 2019[102]). A three kinase cascade core, which activates other MAPKs, is needed for intracellular signaling. Subsequently, this activated MAPKs like p38, JNK1-3, and ERK1/2 regulates numbers of the proteins containing kinase and transcription factors (Fanger, 1999[43]; Prowse and Lew, 2001[126]; Sah et al., 2002[132]; Branca et al., 2004[16]; Hochmann et al., 2016[67]).

The PI3K/AKT pathway is essential for mediating growth and survival in response to extracellular stimuli. PIP3 is generated by phosphorylation of PIP2 by activated PI3K at the plasma membrane. In turn, PDK1 (pyruvate dehydrogenase kinase, isozyme 1) and AKT were recruited to the plasma by PIP3. In this regard, AKT is phosphorylated to its active form by PDK1 (Vara et al., 2004[159]). AKT and MAPK cascade crosswise and in several layers, for decoding and processing extracellular signals, increase extracellular signal expression. The proliferation of various cancers is the consequence of this unnatural action of these pathways. 

The activity of the various effectors for pathways of MAPK is increased via the expression of E6 and E7. Accordingly, the infected cells are modulated across phases of carcinogenesis (e.g., invasion, migration, and Anchorage-independent growth) (Hochmann et al., 2016[67]). Oncogenes derived from the cells are activated and overexpressed via ERK/MAPK in numerous cases of cervical cancers (Mishima et al., 1998[106]; Lessard et al., 2001[95]). The proliferation of HPV related cancers is linked to the regulation of 2 key myogenic signaling pathways including EGFR and VEGF, and by the oncoprotein E5. This protein causes the expression of VEGF through activation of ERK (extracellular signal-regulated kinase). It affects the regulation of the chemical phosphorylation of ERK, in addition to regulating E5, it stabilizes VEGF. The cells contaminated by HPV are protective against autophagy and apoptosis via both E5 protein and the signaling cascades of MAPK-ERK (Kim et al., 2010[86]; DuShane and Maginnis, 2019[39]; DuShane et al., 2019[40]).

### HPV and JAK/STAT pathway

JAK (Janus kinase) -STAT is considered a basic pathway and can regulate the response of the innate immune system (Aaronson, 2002[1]; Reich and Liu, 2006[128]; van Boxel-Dezaire et al., 2006[156]). STAT is characterized as latent cytoplasmic transcription factors that remain in the cytoplasm to become active and then enter the nucleus after activation. STAT family includes different proteins such as STAT-1, -2, -3, -4, -5, and -6. Apoptosis, migration, differentiation as well as the proliferation of cells are the result of JAK activation (Igaz et al., 2001[77]; O'Shea et al., 2002[111]). This pathway is stimulated via several of the corresponding receptors and ligands. Ligand binding further modifies the receptor subunit and activates intracellular activation. The family related to JAK in mammals includes JAK1, JAK2, JAK 3, and Tyk2, which, once activated, targets additional phosphorylate including receptors and main substrates, STATs (Rawlings, 2004[127]). It has been demonstrated that to the conservation of episomes, as well as amplification of genome, it is essential that expression of STAT-1 is suppressed via proteins of HPV (Hong et al., 2011[70]).

In addition, STAT-5 was shown to have a central role in amplification of the HPV-related genome in differentiated cells via induction of the ATM DNA damage pathway. However, the effect of STAT-5 on the conservation of episomes has not been proven in undifferentiated cells (Hong and Laimins, 2013[69]; Bordignon et al., 2017[14]). HPVs need to prevent surveillance of innate and adaptive immune systems to create a persistent infection. The interaction of E6 protein with Tyk2 not only suppresses the phosphorylation of STAT-1/2 and Tyk2 but also hinders the interaction of Tyk2 with the IFN-alpha receptor 1 in the cytoplasmic domain. Consequently, the JAK-STAT pathway is inactivated (Kanodia et al., 2007[83]).

### HPV and YY_1_ pathway

YY1 (Yin and Yang1) belongs to the member of the polycomb group protein family and is recognized as a zinc finger transcription factor (ZFTF). YY1 has a role in epigenetic regulating of transcription, stem-cell identity, disease, and differentiation (Di Croce and Helin, 2013[33]; Hays and Bonavida, 2019[61]). Some are responsible for regulating genes that affect several signaling and progression pathways of cancer like c-fos, E1A, c-myc, ERBB2, and p53. They also physically interact with many proteins that have a regulatory role in apoptosis such as caspase, Mdm2, HDACs, Rb, p53, and Ezh2. Several growth factors can stimulate the expression of the YY1 gene, while antiproliferative signals inhibit its expression (Gordon et al., 2006[54]; Sui, 2009[146]). It acts as a bifunctional transcription factor depending on its bonding and has a stimulatory or inhibitory role in transcription. Besides, YY1 has a significant effect on the LCRs of HPV 16 and 18. Regions of the viral regulatory and multiple cellular functions are regulated by YY1. It contains distinguishable activator and repressor domains (Park, 1995[118]; Lichy, 1996[97]). UCRBP, δ, NF-E1, CF1, NMP-1 are the ubiquitous cellular factor of the YY-1 and have a pivotal function in regulating E6 and E7 oncogenes and HPV infection. YY-1 can function both positively and negatively in viral gene expression (Wang et al., 2006[160]; He et al., 2011[62]; Shishodia et al., 2018[139]). Previous studies have proven that occurring apoptosis in HPV-positive HeLa cells and activation of p53 is a result of the YY1 suppression (He et al., 2011[62]).

### HPV and AP-1 pathway

AP-1 (activator protein-1) is a common phrase for a class of TFs (transcription factors) that was recognized in 1987 which has a function as a DNA binding protein (Schiefer et al., 2015[134]). AP-1 is one of the factors whose transcription activities are controlled by intra and extracellular as well as viral infection (Gazon et al., 2018[52]). Processes of cellular like apoptosis, migration, survival, differentiation, proliferation, and growth of cell are regulated via AP-1 (Schiefer et al., 2015[134]). The type of cell, the stage of the tumor, the genetic history of the tumor, and the cell differentiation condition characterizes the oncogenic or anti-oncogenic AP-1 (Shen et al., 2005[137]) via interaction with the promoter of HPV situated in the upstream regulatory region (URR), which intensifies tumorigenesis and E6 and E7 transcription. In cervical tumors, c-FOS and JUNB are considered as significant parts of dimers related to AP-1 activated during expression of the HPV oncogene (Divya and Pillai, 2006[36]; Chakraborty et al., 2014[22]). Therefore, transcription of HPV is induced. AP-1 is targeted via HPV oncoproteins. Accordingly, the activation of the transcription factor (TF) is induced. For example, expression of E7 which is mediated by c-JUN, and activation of c-JUN are increased via interaction of E7 with the c-JUN (Delcuratolo et al., 2016[30]; Mirzaei et al., 2020[105]).

### HPV and NF-κB pathway

NF-κB (Nuclear Factor Kappa B) is a dimer, with the most common form of p50/p65 heterodimer (Hayden, 2004[59]). In 1986, NF-κB was detected. It acts as a B stem-cell stimulant to bind kappa light-chain (Sen and Baltimore, 1986[136]). NF-κB is considered as a transcription factor that has a pivotal function in immune response, progression/inflammation of cancer, and viral replication of (Hayden et al., 2006[60]; Hoesel and Schmid, 2013[68]). These proteins have several kinds that after phosphorylation and other translational modification organize several homo- or heterodimers that are crucial for translocation, expression, and activation in the nucleus. The binding of NF-κB to the target DNA increases various target genes (Wong et al., 2011[166]; Tilborghs et al., 2017[150]). Binding of the adapter to the cytoplasmic domain of the receptor is usually connected by attaching a ligand to the cell surface receptor, which often adsorbs an IKK complex onto the cytoplasmic adapter and activates the IKK complex. In order to NF-κB entrance into the nucleus to target the genes, IKB must be phosphorylated by activated IKK at the 2 residues of serine and then degradation and ubiquitination of K48 occur via the proteasome (Gilmore, 2006[53]; Perkins, 2006[121]). NF-κB has a negative feedback loop for regulating the increase of HPV that can control the number of virus copies, and control the expression of E6 and E7 proteins in HPV-16 (James et al., 2006[79]; Wong et al., 2011[166]). Immortalization of colonic and colony-forming via HPV-16 is increased by suppression of NF-κB through inhibitor of IκB alpha. Research shows that the control of NF-κB via HPV-16 E6/E7 can prevent cell formation in the cervical region (Vandermark et al., 2012[157]).

### HPV and CXCL12/CXCR4 pathway

CXCL12 (Chemokine ligand (family CXC) 12) is a very important α-chemokine that connects to the G-protein-coupled seven-transmembrane receptor CXCR4 (Chemokine receptor (family CXC) 4) (Bleul et al., 1996[11]; Oberlin et al., 1996[112]). As a result of this binding, a chemokine receptor is activated that relies on Gαi protein-dependent signaling con-trolled by β-arrestins (Busillo and Benovic, 2007[19]). Furthermore, recent studies demonstrated that CXCL12 binds to another receptor CXCR7 sharing this receptor with another chemokine CXCL11 (Maksym et al., 2009[99]). The CXCL12/CXCR4 pathway can activate cell proliferation, cellular migration, cell adhesion, by phosphorylating AKT and several focal adhesion components. It can also cause cellular increase by the Wnt pathway and activation of NF-κB for inhibiting apoptosis (Gu et al., 2014[57]; Wang and Knaut, 2014[161]; Pozzobon et al., 2016[125]). The receptor of CXCL12 is regulated via E6 and E7 proteins, to induce cell and virus proliferation. This can happen in both cases of low or high- risk HPVs, which can eventually cause mutation in CXCR4 and progression of the disease (Busillo and Benovic, 2007[19]; Tommasino, 2014[153]; Okuyama et al., 2016[114]). Expression of E6 and E7 proteins take place in suprabasal layers and then regulates the CXCL12 levels. And this, in turn, causes cell proliferation and an increase in viral DNA in low and high-risk HPVs, which can be amplified by a mutation in CXCR4 (Chapman et al., 2010[24]; Chow et al., 2010[27]; Lecavalier-Barsoum et al., 2018[93]). Furthermore, the half-life and steady-state in E6/E7 proteins are increased as a result of the interaction of CXCR4 with the chaperone proteins (Kuang et al., 2012[90]; Ajiro and Zheng, 2015[2]; Meuris et al., 2016[104]).

## Conclusion

PI3K/Akt, Wnt/β-catenin, ERK/MAPK, NF-κB, YY_1, _AP-1, JAK/STAT and CXCL12/CXCR4 signaling pathways have a significant function in the progression of cervical cancer in HPV infected individuals (Figure 2(Fig. 2)). The development and guidance of each of these pathways toward cancerization is the result of the activity and function of oncoproteins. It appears that there is a meaningful connection between these oncoproteins and the aforementioned pathways that require appropriate tests. It's expected that with an accurate knowledge of these pathways and the factors affecting them, we can achieve our goals in the prevention and efficient cure in this case.

## Notes

Behzad Baradarana and Hossein Bannazadeh Baghi (Department of Virology, Faculty of Medicine, Tabriz University of Medical Sciences, Tabriz, Iran; Tel: +98 41 3336 4661, E-mail: hbannazadeh@tbzmed.ac.ir) equally contributed as corresponding authors.

## Conflict of interest

The authors declare that they have no conflict of interest.

## Funding

This study was financially supported by Immunology Research Center, Tabriz University of Medical Sciences, Tabriz, Iran.

## Acknowledgement

The authors would like to thank the Immunology Research Center, Tabriz University of Medical Sciences for their support.

## Author contributions

F. R. B., B. B., and H. B. B. devised the main conceptual ideas. F. R. B. and M. G. wrote the initial draft of the manuscript. A. B. prepared the figures. N. B., A. M., M. A., K. D., and K. H. reviewed and edited the manuscript. B. B. and H. B. B. supervised the study. All authors of this paper have read and approved the final version submitted.

## Data availability

Data sharing is not applicable to this article as no new data were created or analyzed in this study.

## Figures and Tables

**Table 1:**
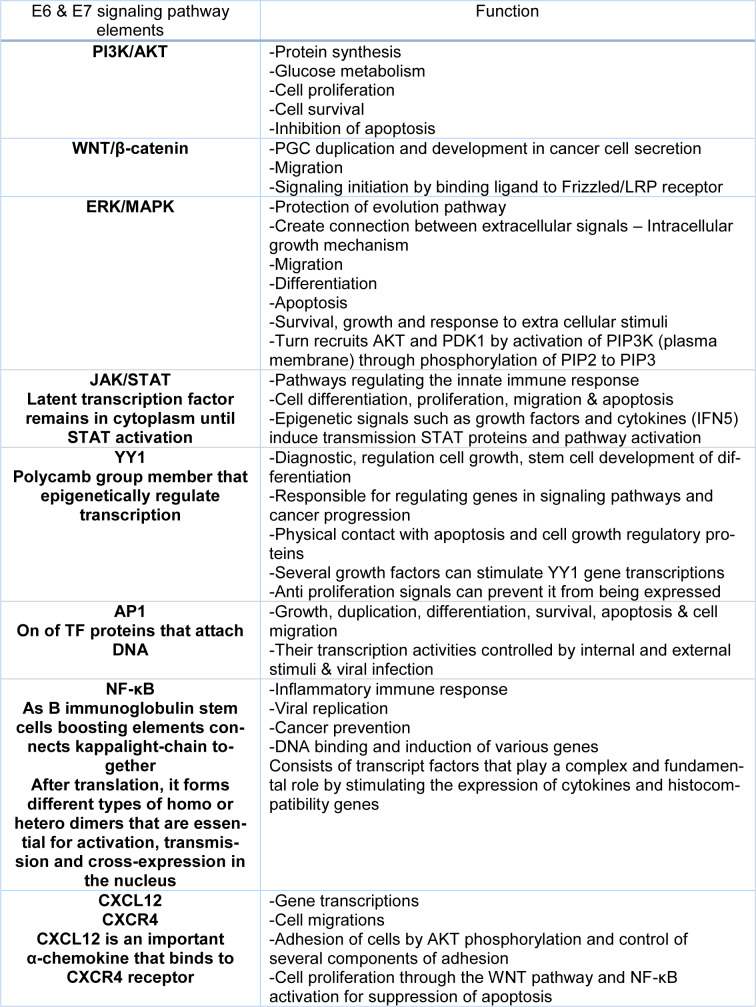
Summary list of some important functions of E6 & E7 signaling pathway elements

## References

[1] Aaronson DS (2002). A road map for those who don’t know JAK-STAT. Science.

[2] Ajiro M, Zheng Z-M (2015). E6^E7, a novel splice isoform protein of human papillomavirus 16, stabilizes viral E6 and E7 oncoproteins via HSP90 and GRP78. mBio.

[3] Ammermann I, Bruckner M, Matthes F, Iftner T, Stubenrauch F (2008). Inhibition of transcription and DNA replication by the papillomavirus E8⁁E2C protein is mediated by interaction with corepressor molecules. J Virol.

[4] Anderson RT, Keysar SB, Bowles DW, Glogowska MJ, Astling DP, Morton JJ (2013). The dual pathway inhibitor rigosertib is effective in direct patient tumor xenografts of head and neck squamous cell carcinomas. Mol Cancer Ther.

[5] Androphy EJ, Hubbert NL, Schiller JT, Lowy DR (1987). Identification of the HPV-16 E6 protein from transformed mouse cells and human cervical carcinoma cell lines. EMBO J.

[6] Banks L, Edmonds C, Vousden KH (1990). Ability of the HPV16 E7 protein to bind RB and induce DNA synthesis is not sufficient for efficient transforming activity in NIH3T3 cells. Oncogene.

[7] Barbosa MS, Edmonds C, Fisher C, Schiller JT, Lowy DR, Vousden KH (1990). The region of the HPV E7 oncoprotein homologous to adenovirus E1a and Sv40 large T antigen contains separate domains for Rb binding and casein kinase II phosphorylation. EMBO J.

[8] Bechtold V, Beard P, Raj K (2003). Human papillomavirus type 16 E2 protein has no effect on transcription from episomal viral DNA. J Virol.

[9] Bello J, Nieva L, Paredes A, Gonzalez A, Zavaleta L, Lizano M (2015). Regulation of the Wnt/β-catenin signaling pathway by human papillomavirus E6 and E7 oncoproteins. Viruses.

[10] Bergvall M, Melendy T, Archambault J (2013). The E1 proteins. Virology.

[11] Bleul CC, Fuhlbrigge RC, Casasnovas JM, Aiuti A, Springer TA (1996). A highly efficacious lymphocyte chemoattractant, stromal cell-derived factor 1 (SDF-1). J Exp Med.

[12] Blodt S, Holmberg C, Muller-Nordhorn J, Rieckmann N (2012). Human Papillomavirus awareness, knowledge and vaccine acceptance: A survey among 18-25 year old male and female vocational school students in Berlin, Germany. Eur J Public Health.

[13] Borbély ÁA, Murvai M, Kónya J, Beck Z, Gergely L, Li F (2006). Effects of human papillomavirus type 16 oncoproteins on survivin gene expression. J Gen Virol.

[14] Bordignon V, Di Domenico E, Trento E, D’Agosto G, Cavallo I, Pontone M (2017). How human papillomavirus replication and immune evasion strategies take advantage of the host DNA damage repair machinery. Viruses.

[15] Bossler F, Kuhn BJ, Günther T, Kraemer SJ, Khalkar P, Adrian S (2019). Repression of human papillomavirus oncogene expression under hypoxia is mediated by PI3K/mTORC2/AKT signaling. mBio.

[16] Branca M, Ciotti M, Santini D, Di Bonito L, Benedetto A, Giorgi C (2004). Activation of the ERK/MAP kinase pathway in cervical intraepithelial neoplasia is related to grade of the lesion but not to high-risk human papillomavirus, virus clearance, or prognosis in cervical cancer. Am J Clin Pathol.

[17] Buck CB, Cheng N, Thompson CD, Lowy DR, Steven AC, Schiller JT (2008). Arrangement of L2 within the Papillomavirus Capsid. J Virol.

[18] Burris HA (2013). Overcoming acquired resistance to anticancer therapy: Focus on the PI3K/AKT/mTOR pathway. Cancer Chemother Pharmacol.

[19] Busillo JM, Benovic JL (2007). Regulation of CXCR4 signaling. Biochim Biophys Acta - Biomembr.

[20] Bzhalava D, Guan P, Franceschi S, Dillner J, Clifford G (2013). A systematic review of the prevalence of mucosal and cutaneous human papillomavirus types. Virology.

[21] Campbell NA, Mitchell LG, Reece JB, Taylor MR (2000). Biology: concepts & connections.

[22] Chakraborty S, Das K, Saha S, Mazumdar M, Manna A, Chakraborty S (2014). Nuclear matrix protein SMAR1 represses c-Fos-mediated HPV18 E6 transcription through alteration of chromatin histone deacetylation. J Biol Chem.

[23] Chang T-H, Liao C-L, Lin Y-L (2006). Flavivirus induces interferon-beta gene expression through a pathway involving RIG-I-dependent IRF-3 and PI3K-dependent NF-κB activation. Microbes Infect.

[24] Chapman S, Liu X, Meyers C, Schlegel R, McBride AA (2010). Human keratinocytes are efficiently immortalized by a Rho kinase inhibitor. J Clin Invest.

[25] Charette ST, McCance DJ (2007). The E7 protein from human papillomavirus type 16 enhances keratinocyte migration in an Akt-dependent manner. Oncogene.

[26] Chawengsaksophak K, Svingen T, Ng ET, Epp T, Spiller CM, Clark C (2012). Loss of Wnt5a disrupts primordial germ cell migration and male sexual development in mice1. Biol Reprod.

[27] Chow KYC, Brotin É, Ben Khalifa Y, Carthagena L, Teissier S, Danckaert A (2010). A pivotal role for CXCL12 Signaling in HPV-Mediated transformation of keratinocytes: Clues to understanding HPV-pathogenesis in WHIM syndrome. Cell Host Microbe.

[28] Costa LG, Giordano G, Guizzetti M, McQueen CAS (2010). Cell signaling and neurotoxicity. Comprehensive toxicology.

[29] de Sanjose S, Quint WG, Alemany L, Geraets DT, Klaustermeier JE, Lloveras B (2010). Human papillomavirus genotype attribution in invasive cervical cancer: A retrospective cross-sectional worldwide study. Lancet Oncol.

[30] Delcuratolo M, Fertey J, Schneider M, Schuetz J, Leiprecht N, Hudjetz B (2016). Papillomavirus-associated tumor formation critically depends on c-Fos expression induced by viral protein E2 and Bromodomain protein Brd4. PLOS Pathog.

[31] DeMasi J, Huh K-W, Nakatani Y, Munger K, Howley PM (2005). Bovine papillomavirus E7 transformation function correlates with cellular p600 protein binding. Proc Natl Acad Sci U S A.

[32] Demers GW, Espling E, Harry JB, Etscheid BG, Galloway DA (1996). Abrogation of growth arrest signals by human papillomavirus type 16 E7 is mediated by sequences required for transformation. J Virol.

[33] Di Croce L, Helin K (2013). Transcriptional regulation by Polycomb group proteins. Nat Struct Mol Biol.

[34] Diehl N, Schaal H (2013). Make yourself at home: Viral hijacking of the PI3K/Akt signaling pathway. Viruses.

[35] DiMaio D, Mattoon D (2001). Mechanisms of cell transformation by papillomavirus E5 proteins. Oncogene.

[36] Divya CS, Pillai MR (2006). Antitumor action of curcumin in human papillomavirus associated cells involves downregulation of viral oncogenes, prevention of NFkB and AP-1 translocation, and modulation of apoptosis. Mol Carcinog.

[37] Doorbar J, Foo C, Coleman N, Medcalf L, Hartley O, Prospero T (1997). Characterization of events during the late stages of HPV16 infectionin vivousing high-affinity synthetic Fabs to E4. Virology.

[38] Dueñas-González A, Lizano M, Candelaria M, Cetina L, Arce C, Cervera E (2005). Epigenetics of cervical cancer. An overview and therapeutic perspectives. Mol Cancer.

[39] DuShane JK, Maginnis MS (2019). Human DNA virus exploitation of the MAPK-ERK cascade. Int J Mol Sci.

[40] DuShane JK, Wilczek MP, Crocker MA, Maginnis MS (2019). High-throughput characterization of viral and cellular protein expression patterns during JC polyomavirus infection. Front Microbiol.

[41] Dyson N, Howley P, Munger K, Harlow E (1989). The human papilloma virus-16 E7 oncoprotein is able to bind to the retinoblastoma gene product. Science.

[42] Egawa N, Nakahara T, Ohno S-i, Narisawa-Saito M, Yugawa T, Fujita M (2012). The E1 protein of human papillomavirus Type 16 is dispensable for maintenance replication of the viral genome. J Virol.

[43] Fanger GR (1999). Regulation of the MAPK family members: role of subcellular localization and architectural organization. Histol Histopathol.

[44] Filippova M, Johnson MM, Bautista M, Filippov V, Fodor N, Tungteakkhun SS (2007). The large and small isoforms of human papillomavirus type 16 E6 bind to and differentially affect procaspase 8 stability and activity. J Virol.

[45] Filippova M, Parkhurst L, Duerksen-Hughes PJ (2004). The human papillomavirus 16 E6 protein binds to fas-associated death domain and protects cells from fas-triggered apoptosis. J Biol Chem.

[46] Filippova M, Song H, Connolly JL, Dermody TS, Duerksen-Hughes PJ (2002). The human papillomavirus 16 E6 protein binds to tumor necrosis factor (TNF) R1 and protects cells from TNF-induced apoptosis. J Biol Chem.

[47] Firzlaff JM, Luscher B, Eisenman RN (1991). Negative charge at the casein kinase II phosphorylation site is important for transformation but not for Rb protein binding by the E7 protein of human papillomavirus type 16. Proc Natl Acad Sci U S A.

[48] Freudenburg W, Moran JM, Lents NH, Baldassare JJ, Buller RML, Corbett JA (2010). Phosphatidylinositol 3-kinase regulates macrophage responses to double-stranded rna and encephalomyocarditis virus. J Innate Immun.

[49] Fu Y, Zheng S, An N, Athanasopoulos T, Popplewell L, Liang A (2011). β-catenin as a potential key target for tumor suppression. Int J Cancer.

[50] Galluzzi L, Brenner C, Morselli E, Touat Z, Kroemer G (2008). Viral control of mitochondrial apoptosis. PLoS Pathog.

[51] Ganguly N, Parihar SP (2009). Human papillomavirus E6 and E7 oncoproteins as risk factors for tumorigenesis. J Biosci.

[52] Gazon H, Barbeau B, Mesnard J-M, Peloponese J-M (2018). Hijacking of the AP-1 signaling pathway during development of ATL. Front Microbiol.

[53] Gilmore TD (2006). Introduction to NF-κB: Players, pathways, perspectives. Oncogene.

[54] Gordon S, Akopyan G, Garban H, Bonavida B (2006). Transcription factor YY1: Structure, function, and therapeutic implications in cancer biology. Oncogene.

[55] Graham SV (2010). Human papillomavirus: gene expression, regulation and prospects for novel diagnostic methods and antiviral therapies. Future Microbiol.

[56] Griesser H, Sander H, Walczak C, Hilfrich RA (2009). HPV vaccine protein L1 predicts disease outcome of high-risk HPV+ early squamous dysplastic lesions. Am J Clin Pathol.

[57] Gu Q, Chen XT, Xiao Bin Y, Chen L, Wang XF, Fang J (2014). Identification of differently expressed genes and small molecule drugs for tetralogy of Fallot by bioinformatics strategy. Pediatr Cardiol.

[58] Gupta S, Kumar P, Das BC (2018). HPV: Molecular pathways and targets. Curr Probl Cancer.

[59] Hayden MS (2004). Signaling to NF-κB. Genes Dev.

[60] Hayden MS, West AP, Ghosh S (2006). NF-κB and the immune response. Oncogene.

[61] Hays E, Bonavida B (2019). YY1 regulates cancer cell immune resistance by modulating PD-L1 expression. Drug Resist Updat.

[62] He G, Wang Q, Zhou Y, Wu X, Wang L, Duru N (2011). YY1 Is a novel potential therapeutic target for the treatment of HPV infection-induced cervical cancer by arsenic trioxide. Int J Gynecol Cancer.

[63] Helt A-M, Galloway DA (2001). Destabilization of the retinoblastoma tumor suppressor by human papillomavirus type 16 E7 is not sufficient to overcome cell cycle arrest in human keratinocytes. J Virol.

[64] Hengstermann A, Linares LK, Ciechanover A, Whitaker NJ, Scheffner M (2001). Complete switch from Mdm2 to human papillomavirus E6-mediated degradation of p53 in cervical cancer cells. Proc Natl Acad Sci U S A.

[65] Herr P, Basler K (2012). Porcupine-mediated lipidation is required for Wnt recognition by Wls. Dev Biol.

[66] Hilger RA, Scheulen ME, Strumberg D (2002). The Ras-Raf-MEK-ERK pathway in the treatment of cancer. Oncol Res Treat.

[67] Hochmann J, Sobrinho JS, Villa LL, Sichero L (2016). The Asian-American variant of human papillomavirus type 16 exhibits higher activation of MAPK and PI3K/AKT signaling pathways, transformation, migration and invasion of primary human keratinocytes. Virology.

[68] Hoesel B, Schmid JA (2013). The complexity of NF-κB signaling in inflammation and cancer. Mol Cancer.

[69] Hong S, Laimins LA (2013). The JAK-STAT Transcriptional regulator, STAT-5, activates the ATM DNA damage pathway to induce HPV 31 genome amplification upon epithelial differentiation. PLoS Pathog.

[70] Hong S, Mehta KP, Laimins LA (2011). Suppression of STAT-1 expression by human papillomaviruses is necessary for differentiation-dependent genome amplification and plasmid maintenance. J Virol.

[71] Hoppe-Seyler K, Bossler F, Lohrey C, Bulkescher J, Rösl F, Jansen L (2017). Induction of dormancy in hypoxic human papillomavirus-positive cancer cells. Proc Natl Acad Sci U S A.

[72] Hoque E, Hoque M (2009). Knowledge of and attitude towards cervical cancer among female university students in South Africa. S Afr J Epidemiol Infect.

[73] Hou SY, Wu S-Y, Zhou T, Thomas MC, Chiang C-M (2000). Alleviation of human papillomavirus E2-mediated transcriptional repression via formation of a TATA binding protein (or TFIID)-TFIIB-RNA polymerase II-TFIIF preinitiation complex. Mol Cell Biol.

[74] Huang S-M, McCance DJ (2002). Down regulation of the interleukin-8 promoter by human papillomavirus type 16 E6 and E7 through effects on CREB binding protein/p300 and P/CAF. J Virol.

[75] Huh K, Zhou X, Hayakawa H, Cho J-Y, Libermann TA, Jin J (2007). Human papillomavirus type 16 E7 oncoprotein associates with the cullin 2 ubiquitin ligase complex, which contributes to degradation of the retinoblastoma tumor suppressor. J Virol.

[76] Huh K-W, DeMasi J, Ogawa H, Nakatani Y, Howley PM, Munger K (2005). Association of the human papillomavirus type 16 E7 oncoprotein with the 600-kDa retinoblastoma protein-associated factor, p600. Proc Natl Acad Sci U S A.

[77] Igaz P, Tóth S, Falus A (2001). Biological and clinical significance of the JAK-STAT pathway;lessons from knockout mice. Inflamm Res.

[78] Jackson S, Storey A (2000). E6 proteins from diverse cutaneous HPV types inhibit apoptosis in response to UV damage. Oncogene.

[79] James MA, Lee JH, Klingelhutz AJ (2006). Human papillomavirus type 16 E6 activates NF-κB, induces cIAP-2 expression, and protects against apoptosis in a PDZ binding motif-dependent manner. J Virol.

[80] Jemal A, Bray F, Center MM, Ferlay J, Ward E, Forman D (2011). Global cancer statistics. CA Cancer J Clin.

[81] Jones DL, Thompson DA, Münger K (1997). Destabilization of the RB tumor suppressor protein and stabilization of p53 contribute to HPV type 16 E7-induced apoptosis. Virology.

[82] Kang J, Rychahou PG, Ishola TA, Mourot JM, Evers BM, Chung DH (2008). N-myc is a novel regulator of PI3K-mediated VEGF expression in neuroblastoma. Oncogene.

[83] Kanodia S, Fahey L, Kast WM (2007). Mechanisms used by human papillomaviruses to escape the host immune response. Curr Cancer Drug Targets.

[84] Kaur S, Katsoulidis E, Platanias LC (2008). Akt and mRNA translation by interferons. Cell Cycle.

[85] Kim K, Lambert PF (2002). E1 protein of bovine papillomavirus 1 is not required for the maintenance of viral plasmid DNA replication. Virology.

[86] Kim M-K, Kim HS, Kim S-H, Oh J-M, Han JY, Lim JM (2010). Human papillomavirus type 16 E5 oncoprotein as a new target for cervical cancer treatment. Biochem Pharmacol.

[87] Kimura T, Nakamura T, Murayama K, Umehara H, Yamano N, Watanabe S (2006). The stabilization of β-catenin leads to impaired primordial germ cell development via aberrant cell cycle progression. Dev Biol.

[88] Kontostathi G, Zoidakis J, Anagnou NP, Pappa KI, Vlahou A, Makridakis M (2016). Proteomics approaches in cervical cancer: focus on the discovery of biomarkers for diagnosis and drug treatment monitoring. Expert Rev Proteomics.

[89] Kruiswijk F, Labuschagne CF, Vousden KH (2015). p53 in survival, death and metabolic health: a lifeguard with a licence to kill. Nat Rev Mol Cell Biol.

[90] Kuang Y-Q, Charette N, Frazer J, Holland PJ, Attwood KM, Dellaire G (2012). Dopamine receptor-interacting protein 78 acts as a molecular chaperone for CCR5 chemokine receptor signaling complex organization. PLoS One.

[91] Kuslansky Y, Sominsky S, Jackman A, Gamell C, Monahan BJ, Haupt Y (2016). Ubiquitin ligase E6AP mediates nonproteolytic polyubiquitylation of β-catenin independent of the E6 oncoprotein. J Gen Virol.

[92] Laird DJ, Altshuler-Keylin S, Kissner MD, Zhou X, Anderson KV (2011). Ror2 enhances polarity and directional migration of primordial germ cells. PLoS Genet.

[93] Lecavalier-Barsoum M, Chaudary N, Han K, Koritzinsky M, Hill R, Milosevic M (2018). Targeting the CXCL12/CXCR4 pathway and myeloid cells to improve radiation treatment of locally advanced cervical cancer. Int J Cancer.

[94] Lee HC, Lim S, Han JY (2016). Wnt/β-catenin signaling pathway activation is required for proliferation of chicken primordial germ cells in vitro. Sci Rep.

[95] Lessard JL, Robinson RA, Hoffman HT (2001). Differential expression of ras signal transduction mediators in verrucous and squamous cell carcinomas of the upper aerodigestive tract. Arch Pathol Lab Med.

[96] Li H, Jiao S, Li X, Banu H, Hamal S, Wang X (2015). Therapeutic effects of antibiotic drug tigecycline against cervical squamous cell carcinoma by inhibiting Wnt/β-catenin signaling. Biochem Biophys Res Commun.

[97] Lichy J (1996). Differential expression of the human ST5 gene in HeLa-fibroblast hybrid cell lines mediated by YY1: evidence that YY1 plays a part in tumor suppression. Nucleic Acids Res.

[98] Lopez J, Tait SWG (2015). Mitochondrial apoptosis: Killing cancer using the enemy within. Br J Cancer.

[99] Maksym RB, Tarnowski M, Grymula K, Tarnowska J, Wysoczynski M, Liu R (2009). The role of stromal-derived factor-1 — CXCR7 axis in development and cancer. Eur J Pharmacol.

[100] Manikandan S, Behera S, Naidu N, Angamuthu V, Mohammed OB, Debata A (2019). Knowledge and awareness toward cervical cancer screening and prevention among the professional college female students. J Pharm Bioallied Sci.

[101] Manning BD, Toker A (2017). AKT/PKB signaling: Navigating the network. Cell.

[102] Mansoori B, Mohammadi A, Ghasabi M, Shirjang S, Dehghan R, Montazeri V (2019). miR‐142‐3p as tumor suppressor miRNA in the regulation of tumorigenicity, invasion and migration of human breast cancer by targeting Bach‐1 expression. J Cell Physiol.

[103] Mesri EA, Feitelson MA, Munger K (2014). Human viral oncogenesis: A cancer hallmarks analysis. Cell Host Microbe.

[104] Meuris F, Carthagena L, Jaracz-Ros A, Gaudin F, Cutolo P, Deback C (2016). The CXCL12/CXCR4 signaling pathway: A new susceptibility factor in human papillomavirus pathogenesis. PLOS Pathog.

[105] Mirzaei H, Khodadad N, Karami C, Pirmoradi R, Khanizadeh S (2020). The AP‐1 pathway;A key regulator of cellular transformation modulated by oncogenic viruses. Rev Med Virol.

[106] Mishima K, Yamada E, Masui K, Shimokawara T, Takayama K, Sugimura M (1998). Overexpression of the ERK/MAP kinases in oral squamous cell carcinoma. Mod Pathol.

[107] Nakatani Y, Konishi H, Vassilev A, Kurooka H, Ishiguro K, Sawada J-i (2005). p600, a unique protein required for membrane morphogenesis and cell survival. Proc Natl Acad Sci U S A.

[108] Nour NM (2009). Cervical cancer: a preventable death. Rev Obstet Gynecol.

[109] Nusse R (2005). Wnt signaling in disease and in development. Cell Res.

[110] Nusse R, Clevers H (2017). Wnt/β-catenin signaling, disease, and emerging therapeutic modalities. Cell.

[111] O’Shea JJ, Gadina M, Schreiber RD (2002). Cytokine signaling in 2002. Cell.

[112] Oberlin E, Amara A, Bachelerie F, Bessia C, Virelizier J-L, Arenzana-Seisdedos F (1996). The CXC chemokine SDF-1 is the ligand for LESTR/fusin and prevents infection by T-cell-line-adapted HIV-1. Nature.

[113] Ohinata Y, Ohta H, Shigeta M, Yamanaka K, Wakayama T, Saitou M (2009). A signaling principle for the specification of the germ cell lineage in mice. Cell.

[114] Okuyama NCM, dos Santos FC, Trugilo KP, Brajão de Oliveira K (2016). Involvement of CXCL12 pathway in HPV-related diseases. AIMS Med Sci.

[115] Ozbun MA (2002). Human papillomavirus type 31b infection of human keratinocytes and the onset of early transcription. J Virol.

[116] Paek AL, Liu JC, Loewer A, Forrester WC, Lahav G (2016). Cell-to-cell variation in p53 dynamics leads to fractional killing. Cell.

[117] Pappa KI, Lygirou V, Kontostathi G, Zoidakis J, Makridakis M, Vougas K (2017). Proteomic analysis of normal and cancer cervical cell lines reveals deregulation of cytoskeleton-associated proteins. Cancer Genomics Proteomics.

[118] Park K (1995). Characterization of functional domains within the multifunctional transcription factor, YY1. J Biol Chem.

[119] Patel D (1999). The E6 protein of human papillomavirus type 16 binds to and inhibits co-activation by CBP and p300. EMBO J.

[120] Patrick DR, Oliff A, Heimbrook DC (1994). Identification of a novel retinoblastoma gene product binding site on human papillomavirus type 16 E7 protein. J Biol Chem.

[121] Perkins ND (2006). Post-translational modifications regulating the activity and function of the nuclear factor kappa B pathway. Oncogene.

[122] Pim D, Massimi P, Dilworth SM, Banks L (2005). Activation of the protein kinase B pathway by the HPV-16 E7 oncoprotein occurs through a mechanism involving interaction with PP2A. Oncogene.

[123] Pim D, Storey A, Thomas M, Massimi P, Banks L (1994). Mutational analysis of HPV-18 E6 identifies domains required for p53 degradation in vitro, abolition of p53 transactivation in vivo and immortalisation of primary BMK cells. Oncogene.

[124] Polakis P (2012). Wnt signaling in cancer. Cold Spring Harb Perspect Biol.

[125] Pozzobon T, Goldoni G, Viola A, Molon B (2016). CXCR4 signaling in health and disease. Immunol Lett.

[126] Prowse CN, Lew J (2001). Mechanism of activation of ERK2 by dual phosphorylation. J Biol Chem.

[127] Rawlings JS (2004). The JAK/STAT signaling pathway. J Cell Sci.

[128] Reich NC, Liu L (2006). Tracking STAT nuclear traffic. Nat Rev Immunol.

[129] Roy F (2002). KSR is a scaffold required for activation of the ERK/MAPK module. Genes Dev.

[130] Rudin CM, Thompson CB (1997). Apoptosis and disease: Regulation and clinical relevance of programmed cell death. Annu Rev Med.

[131] Ruttkay-Nedecky B, Jimenez Jimenez AM, Nejdl L, Chudobova D, Gumulec J, Masarik M (2013). Relevance of infection with human papillomavirus: the role of the p53 tumor suppressor protein and E6/E7 zinc finger proteins. Int J Oncol.

[132] Sah JF, Eckert RL, Chandraratna RAS, Rorke EA (2002). Retinoids suppress epidermal growth factor-associated cell proliferation by inhibiting epidermal growth factor receptor-dependent ERK1/2 activation. J Biol Chem.

[133] Sato N, Meijer L, Skaltsounis L, Greengard P, Brivanlou AH (2004). Maintenance of pluripotency in human and mouse embryonic stem cells through activation of Wnt signaling by a pharmacological GSK-3-specific inhibitor. Nat Med.

[134] Schiefer A-I, Vesely P, Hassler MR, Egger G, Kenner L (2015). The role of AP-1 and epigenetics in ALCL. Front Biosci.

[135] Schwarz E, Freese UK, Gissmann L, Mayer W, Roggenbuck B, Stremlau A (1985). , Structure and transcription of human papillomavirus sequences in cervical carcinoma cells. Nature.

[136] Sen R, Baltimore D (1986). Inducibility of κ immunoglobulin enhancer-binding protein NF-κB by a posttranslational mechanism. Cell.

[137] Shen G, Jeong W-S, Hu R, Kong A-NT (2005). Regulation of Nrf2, NF-κB, and AP-1 Signaling Pathways by Chemopreventive Agents. Antioxid Redox Signal.

[138] Shirjang S, Mansoori B, Asghari S, Duijf PHG, Mohammadi A, Gjerstorff M (2019). MicroRNAs in cancer cell death pathways: Apoptosis and necroptosis. Free Radic Biol Med.

[139] Shishodia G, Verma G, Das BC, Bharti AC (2018). miRNA as viral transcription tuners in HPV-mediated cervical carcinogenesis. Front Biosci (Schol Ed).

[140] Smith JS, Lindsay L, Hoots B, Keys J, Franceschi S, Winer R (2007). Human papillomavirus type distribution in invasive cervical cancer and high-grade cervical lesions: A meta-analysis update. Int J Cancer.

[141] Sominsky S, Kuslansky Y, Shapiro B, Jackman A, Haupt Y, Rosin-Arbesfeld R (2014). HPV16 E6 and E6AP differentially cooperate to stimulate or augment Wnt signaling. Virology.

[142] Sominsky S, Shterzer N, Jackman A, Shapiro B, Yaniv A, Sherman L (2017). E6 proteins of α and β cutaneous HPV types differ in their ability to potentiate Wnt signaling. Virology.

[143] Spangle JM, Münger K (2010). The human papillomavirus type 16 E6 oncoprotein activates mTORC1 signaling and increases protein synthesis. J Virol.

[144] Stanley M (2006). Immune responses to human papillomavirus. Vaccine.

[145] Stubenrauch F, Hummel M, Iftner T, Laimins LA (2000). The E8^E2C protein, a negative regulator of viral transcription and replication, is required for extrachromosomal maintenance of human papillomavirus type 31 in keratinocytes. J Virol.

[146] Sui G (2009). The regulation of YY1 in tumorigenesis and its targeting potential in cancer therapy. Mol Cell Pharmacol.

[147] Thomas M, Banks L (1999). Human papillomavirus (HPV) E6 interactions with Bak are conserved amongst E6 proteins from high and low risk HPV types. J Gen Virol.

[148] Thomas M, Banks L (1998). Inhibition of Bak-induced apoptosis by HPV-18 E6. Oncogene.

[149] Thomas MC, Chiang C-M (2005). E6 oncoprotein represses p53-dependent gene activation via inhibition of protein acetylation independently of inducing p53 degradation. Mol Cell.

[150] Tilborghs S, Corthouts J, Verhoeven Y, Arias D, Rolfo C, Trinh XB (2017). The role of nuclear factor-kappa B signaling in human cervical cancer. Crit Rev Oncol Hematol.

[151] Tindle RW (2002). Immune evasion in human papillomavirus-associated cervical cancer. Nat Rev Cancer.

[152] Tomaić V (2016). Functional roles of E6 and E7 oncoproteins in HPV-induced malignancies at diverse anatomical sites. Cancers (Basel).

[153] Tommasino M (2014). The human papillomavirus family and its role in carcinogenesis. Semin Cancer Biol.

[154] Um SH, Mundi N, Yoo J, Palma DA, Fung K, MacNeil D (2014). Variable expression of the forgotten oncogene E5 in HPV-positive oropharyngeal cancer. J Clin Virol.

[155] Valenta T, Hausmann G, Basler K (2012). The many faces and functions of β-catenin. EMBO J.

[156] van Boxel-Dezaire AHH, Rani MRS, Stark GR (2006). Complex modulation of cell type-specific signaling in response to type I interferons. Immunity.

[157] Vandermark ER, Deluca KA, Gardner CR, Marker DF, Schreiner CN, Strickland DA (2012). Human papillomavirus type 16 E6 and E 7 proteins alter NF-kB in cultured cervical epithelial cells and inhibition of NF-kB promotes cell growth and immortalization. Virology.

[158] Vanhaesebroeck B, Stephens L, Hawkins P (2012). PI3K signalling: The path to discovery and understanding. Nat Rev Mol Cell Biol.

[159] Vara JÁF, Casado E, de Castro J, Cejas P, Belda-Iniesta C, González-Barón M (2004). PI3K/Akt signalling pathway and cancer. Cancer Treat Rev.

[160] Wang C-C, Chen JJ, Yang P-C (2006). Multifunctional transcription factor YY1: A therapeutic target in human cancer?. Expert Opin Ther Targets.

[161] Wang J, Knaut H (2014). Chemokine signaling in development and disease. Development.

[162] Wechsler EI, Tugizov S, Herrera R, Da Costa M, Palefsky JM (2018). E5 can be expressed in anal cancer and leads to epidermal growth factor receptor-induced invasion in a human papillomavirus 16-transformed anal epithelial cell line. J Gen Virol.

[163] White EA, Kramer RE, Tan MJA, Hayes SD, Harper JW, Howley PM (2012). Comprehensive analysis of host cellular interactions with human papillomavirus e6 proteins identifies new E6 binding partners and reflects viral diversity. J Virol.

[164] Whitman M, Kaplan DR, Schaffhausen B, Cantley L, Roberts TM (1985). Association of phosphatidylinositol kinase activity with polyoma middle-T competent for transformation. Nature.

[165] Willert K, Brown JD, Danenberg E, Duncan AW, Weissman IL, Reya T (2003). Wnt proteins are lipid-modified and can act as stem cell growth factors. Nature.

[166] Wong D, Teixeira A, Oikonomopoulos S, Humburg P, Lone I, Saliba D (2011). Extensive characterization of NF-κB binding uncovers non-canonical motifs and advances the interpretation of genetic functional traits. Genome Biol.

[167] Yim E-K, Park J-S (2005). The role of HPV E6 and E7 oncoproteins in HPV-associated cervical carcinogenesis. Cancer Res Treat.

[168] Yousefi B, Darabi M, Baradaran B, Khaniani MS, Rahbani M, Darabi M (2012). Inhibition of MEK/ ERK1/2 signaling affects the fatty acid composition of HepG2 human hepatic cell line. BioImpacts.

[169] Zhang B, Song Y, Sun S, Han R, Hua C, van der Veen S (2019). Human papillomavirus 11 early protein E6 activates autophagy by repressing AKT/mTOR and Erk/mTOR. J Virol.

[170] Zheng Z-M, Baker CC (2006). Papillomavirus genome structure, expression, and post-transcriptional regulation. Front Biosci.

[171] Zimmerli D, Cecconi V, Valenta T, Hausmann G, Cantù C, Restivo G (2018). WNT ligands control initiation and progression of human papillomavirus-driven squamous cell carcinoma. Oncogene.

[172] Zimmerli D, Hausmann G, Cantù C, Basler K (2017). Pharmacological interventions in the Wnt pathway: inhibition of Wnt secretion versus disrupting the protein-protein interfaces of nuclear factors. Br J Pharmacol.

[173] Zimmermann H, Degenkolbe R, Bernard H-U, O’Connor MJ (1999). The human papillomavirus type 16 E6 oncoprotein can down-regulate p53 activity by targeting the transcriptional coactivator CBP/p300. J Virol.

